# Analgesic Effects of Lipid Raft Disruption by Sphingomyelinase and Myriocin via Transient Receptor Potential Vanilloid 1 and Transient Receptor Potential Ankyrin 1 Ion Channel Modulation

**DOI:** 10.3389/fphar.2020.593319

**Published:** 2021-01-27

**Authors:** Ádám Horváth, Maja Payrits, Anita Steib, Boglárka Kántás, Tünde Biró-Süt, János Erostyák, Géza Makkai, Éva Sághy, Zsuzsanna Helyes, Éva Szőke

**Affiliations:** ^1^ Deparment of Pharmacology and Pharmacotherapy, University of Pécs, Medical School, Pécs, Hungary; ^2^ János Szentágothai Research Centre and Centre for Neuroscience, University of Pécs, Pécs, Hungary; ^3^ Department of Experimental Physics, Faculty of Sciences, University of Pécs, Pécs, Hungary; ^4^ Department of Pharmacology and Pharmacotherapy, Semmelweis University, Budapest, Hungary

**Keywords:** lipid raft, myriocin, pain, sensory neuron, sphingomyelinase, transient receptor potential

## Abstract

Transient Receptor Potential (TRP) Vanilloid 1 and Ankyrin 1 (TRPV1, TRPA1) cation channels are expressed in nociceptive primary sensory neurons, and integratively regulate nociceptor and inflammatory functions. Lipid rafts are liquid-ordered plasma membrane microdomains rich in cholesterol, sphingomyelin and gangliosides. We earlier showed that lipid raft disruption inhibits TRPV1 and TRPA1 functions in primary sensory neuronal cultures. Here we investigated the effects of sphingomyelinase (SMase) cleaving membrane sphingomyelin and myriocin (Myr) prohibiting sphingolipid synthesis in mouse pain models of different mechanisms. SMase (50 mU) or Myr (1 mM) pretreatment significantly decreased TRPV1 activation (capsaicin)-induced nocifensive eye-wiping movements by 37 and 41%, respectively. Intraplantar pretreatment by both compounds significantly diminished TRPV1 stimulation (resiniferatoxin)-evoked thermal allodynia developing mainly by peripheral sensitization. SMase (50 mU) also decreased mechanical hyperalgesia related to both peripheral and central sensitizations. SMase (50 mU) significantly reduced TRPA1 activation (formalin)-induced acute nocifensive behaviors by 64% in the second, neurogenic inflammatory phase. Myr, but not SMase altered the plasma membrane polarity related to the cholesterol composition as shown by fluorescence spectroscopy. These are the first *in vivo* results showing that sphingolipids play a key role in lipid raft integrity around nociceptive TRP channels, their activation and pain sensation. It is concluded that local SMase administration might open novel perspective for analgesic therapy.

## Introduction

Cell physiology is influenced by lipid rafts, which are specialized cholesterol-, sphingomyelin- and ganglioside-rich plasma membrane microdomains, surrounding several receptors and ion channels (Simons and Ikonen, 1997). Membrane disintegration causes pathophysiological processes, such as neurodegeneration, neuropathy (Lee et al., 2014; Sonnino et al., 2014; Gambert et al., 2017) and synaptic transmission disruption (De Chiara et al., 2013). Modifying plasma membrane functions is a mechanism of action for pharmacological therapeutic interventions and/or side effects like the cytotoxic actions of anticancer drugs (Adinolfi et al., 2013), endocannabinoid-mediated analgesia (Rossi et al., 2012) and hyperalgesia (Dina et al., 2005). Our previous studies provided evidence that lipid raft disruption decreased the activation of Transient Receptor Potential (TRP) ion channels. These receptors are nonselective cation channels that open in response to temperature changes, binding of a broad range of exogenous and endogenous ligands, as well as other alterations of the channel protein (Gees et al., 2012; Vay et al., 2012). TRP “channelopathies” induce several diseases, such as skeletal muscle disorders, multiple kidney diseases and inherited pain syndrome (Moran et al., 2011; Nilius and Szallasi, 2014). Although pharmaceutical companies have put great efforts and investments into the development of TRP antagonists, only few drug candidates have reached the clinical stages of drug development (Szolcsányi and Sándor, 2012; Kaneko and Szallasi, 2014; Nilius and Szallasi, 2014).

TRP Vanilloid 1 (TRPV1) - expressed in the large population of polymodal nociceptors - is a nocisensor plasma protein gated by noxious heat (>43°C), protons (pH < 6.0), vanilloid-type agonists such as capsaicin (CAPS) and its synthetic analogs, resiniferatoxin (RTX), endogenous arachidonic acid or other fatty acid metabolites (Hwang et al., 2000; Smart et al., 2000; Welch et al., 2000; Raisinghani et al., 2005; Bianchi et al., 2006; Caterina and Park, 2006; Gavva, 2008; Myers et al., 2008; Szolcsányi, 2008; Cao et al., 2013; Aiello et al., 2016). Another similar TRP channel, TRP Ankyrin 1 (TRPA1), is often colocalized with TRPV1 on the CAPS-sensitive sensory nerves (Salas et al., 2009). Exogenous irritants, such as allyl-isothiocyanate (in mustard oil), cinnamaldehyde, allicin, 4-hydroxynonenal and mediators produced by inflammation or tissue injury, e.g., formaldehyde and methylglyoxal, as well as cold (below 17°C) and mechanical stimuli activate the TRPA1 receptor (Story et al., 2003; Bandell et al., 2004; Corey et al., 2004; Jordt et al., 2004; Macpherson et al., 2005, Macpherson et al., 2007; McNamara et al., 2007; Trevisani et al., 2007; Vilceanu and Stucky, 2010; Bautista et al., 2013; De Logu et al., 2019). Recent studies showed that both channels have some interactions with the endocannabinoid system and play an integrative role in regulating nociceptor and inflammatory functions (Akopian et al., 2008; Salas et al., 2009; Brizzi et al., 2014; Marrone et al., 2017). Pro-inflammatory neuropeptides, such as Substance P and calcitonin gene-related peptide (CGRP) released from the CAPS-sensitive fibres induce vasodilation, plasma protein extravasation and inflammatory cell activation (neurogenic inflammation) in the innervated area (Helyes et al., 2003, Helyes et al., 2009; Szolcsányi, 2004).

TRP channels are surrounded by lipid rafts of the plasma membrane modifying their functions (Liu et al., 2006; Morenilla-Palao et al., 2009; Szőke et al., 2010; Sághy et al., 2015). However, data are controversial about the outcomes of lipid raft disruption on TRP channel functions. Impaired TRP Canonical 1 (TRPC1) and TRP Canonical 3 (TRPC3) signaling was reported after methyl β-cyclodextrin (MCD) incubation depleting membrane cholesterol (Lockwich et al., 2000; Bergdahl et al., 2003; Graziani et al., 2006). In dorsal root ganglion neurons MCD treatment significantly reduced the CAPS-activated currents (Liu et al., 2006), but it had no effect on heat-evoked responses on TRPV1-transfected *Xenopus laevis* oocytes (Liu et al., 2003). MCD did not influence ^3^[H]RTX binding to TRPV1 receptors on rat C6 glioma cells (Bari et al., 2005). Sphingomyelinase (SMase) hydrolyzes sphingomyelin (SM) to phosphocholine and ceramide (Kiyokawa et al., 2005; Kobayashi et al., 2006), thus influences the signaling through the cell membrane (Chao et al., 2011). The third mechanism to disrupt the lipid rafts besides MCD and SMase is to block the sphingolipid synthesis by inhibiting of serine palmitoyltransferase by myriocin (Myr) (Miyake et al., 1995).

Our previous results suggested that disrupting lipid rafts by pharmacologically depleting their various constituents, such as SM, cholesterol or gangliosides inhibited the CAPS-, and RTX-induced opening properties of TRPV1 and TRPA1 both on native sensory neurons and receptor-expressing cell lines (Szőke et al., 2010; Sághy et al., 2015). Fluorescence spectroscopy and filipin staining clearly supported the ability of MCD to deplete cholesterol from the cell membrane (Sághy et al., 2015). Besides the sensory neuronal cell bodies we also showed the ability of SMase to diminish TRPV1 and TRPA1 activation on the nerve terminals by measuring the release of the neuropeptide CGRP (Sághy et al., 2015).

Despite all these *in vitro* data showing that lipid raft disruption inhibits TRP channel activation, there are only few very recent reports investigating this phenomenon *in vivo*. Hyperalgesia responses in the RTX-evoked mouse neuropathy model (Lin et al., 2019) and prostaglandin E2 (PGE2) (Ferrari and Levine, 2015) administration were significantly attenuated by MCD. We recently reported the antihyperalgesic actions of MCD and a novel carboxamido-steroid compound in TRPV1 and TRPA1 activation-related mouse pain models (Horváth et al., 2020). Furthermore Myr exerted antitumor activity in a mouse melanoma model (Lee et al., 2011, Lee et al., 2012).

The aim of the present study is to examine the effects of SMase and Myr in mouse pain models of different mechanisms related to TRPV1 and TRPA1 activation.

## Materials and Methods

### Ethics and Animals

Twelve- to Sixteen-week-old male NMRI mice were used in the formalin and RTX tests and male C57BL/6 mice of the same age in the CAPS-evoked wiping test. The animals were kept in the Laboratory Animal House of the Department of Pharmacology and Pharmacotherapy, University of Pécs. All experimental procedures were carried out according to the 1998/XXVIII Act of the Hungarian Parliament on Animal Protection and Consideration Decree of Scientific Procedures of Animal Experiments (243/1988). The studies were approved by the Ethics Committee on Animal Research of Pécs University according to the Ethical Codex of Animal Experiments and license was given (license no. BAI/35/702-6/2018).

### Capsaicin-Evoked Acute Chemonocifensive Reaction

To characterize the effect of SMase and Myr - compared to the saline or dimethyl sulfoxide (DMSO) controls - on acute chemonociception, 30 µg/ml CAPS (20 μl, TRPV1 agonist) was instilled in the right eye of mice. Local pretreatments (20 µl) with 50 mU SMase or 1 mM Myr were performed 30 min or 24 h before the examination, respectively. CAPS-induced eye-wiping movements with the forelegs were counted in a 1-min period, as previously described (Szolcsányi et al., 1975; Szöke et al., 2002; Horváth et al., 2020). Only the one-leg movements were counted, washing-, or two-hand movements were not considered. CAPS administration was repeated in the second and third hours of the test.

### Resiniferatoxin-Induced Thermal Allodynia and Mechanical Hyperalgesia

The effects of SMase and Myr were compared to the saline or DMSO controls in the RTX-induced (ultrapotent TRPV1 agonist) thermal allodynia and mechanonociceptive hyperalgesia model. RTX (0.1 µg/ml, 20 µl) was injected into right hindpaw of the animals, which evokes acute neurogenic inflammatory response with thermal allodynia and mechanical hyperalgesia (Meyer and Campbell, 1981; Pan et al., 2003). Baseline thermal- and mechanical threshold values were determined on two consecutive days, and these data were used for self-control comparisons. Intraplantar pretreatments (20 µl) with 50 mU SMase or 1 mM Myr were performed 30 min or 24 h before the RTX injection, respectively. RTX injection evoked an acute nocifensive reaction of paw licking, biting, lifting or shaking, but these behavioral changes lasted for less than a couple of minutes. The thermonociceptive threshold was measured by an increasing temperature Hot Plate (IITC Life Science, Woodland Hills, CA, United States) in the 10th, 20th and 30th min (Almási et al., 2003; Kántás et al., 2019; Horváth et al., 2020), and the mechanonociceptive threshold by a Dynamic Plantar Aesthesiometer (DPA, Ugo Basile, Italy) in the 30th, 60th and 90th min, as described earlier (Payrits et al., 2017; Kántás et al., 2019; Horváth et al., 2020).

### Formalin-Evoked Acute Nocifensive Behavior

The effect of SMase and Myr compared to the saline- or DMSO-pretreated controls, were investigated on formalin-evoked (20 μl, 2.5%; into the right hindpaw) nocifensive behaviors. Intraplantar pretreatments (20 µl) with 50 mU SMase or 1 mM Myr were performed 30 min and 24 h before the examination, respectively. The duration of the nocifensive behaviors (hind paw licking, biting, shaking and holding) was monitored in two phase (0–5 min and 20–45 min) (Bölcskei et al., 2005; Horváth et al., 2020). The first phase is related to the direct chemical stimulation of nociceptors, e.g., TRPA1. There is a period of 10–15 min, when the animals show lack of nocifensive reactions. The second phase starts 15–20 min after the formalin injection, and it is referring to neurogenic inflammatory mechanisms. For more details see work of Tjolsen and co-workers (Tjølsen et al., 1992).

### Fluorescence Spectroscopy to Determine Membrane Polarity Related to Lipid Raft Integrity

Native Chinese Hamster Ovary (CHO) cells were incubated with 6-dodecanoyl-N,N-dimethyl-2-naphthylamine (Laurdan) in 40 µM final concentration for 40 min at 37°C in a humidified atmosphere with 5% CO_2_. The spectral shape and position of fluorescence emission and excitation spectra of Laurdan depend both on the speed of its dipolar relaxation and the polarity of its microenvironment in the membrane (Harris et al., 2002; Gaus et al., 2003). Cells were treated with 30 mU SMase or 100 nM Myr – dissolved in extracellular solution (ECS) - for 45 min at 37°C before Laurdan administration, then washed three times with phosphate-buffered saline (PBS) and scraped from the plates into 1 ml PBS.

Fluorescence excitation and emission spectra, excitation-emission matrices and anisotropy spectra, were measured by a HORIBA Jobin-Yvon Nanolog FL3-2iHR spectrofluorometer equipped with a 450-W xenon lamp. Samples were measured in a 4 mm path length quartz cuvette (Hellma 104F-QS) and kept at a constant 20°C using a Thermo Scientific circulating bath AC200-A25. Excitation-emission matrices consisting of a series of emission spectra recorded at different excitation wavelengths were measured to determine spectral changes. An excitation-emission matrix has one axis for the emission wavelengths, while the other includes the excitation wavelengths. At the intersection points, fluorescence intensity can be read as the value of the third axis. Steady-state emission anisotropy was measured in “L-format” arrangements to study the molecular mobility. Excitation was vertically polarized, while anisotropy was calculated from consecutively measured vertical and horizontally polarized emission intensities. Anisotropy <*r*> is defined as:

$$\langle r\rangle =\frac{I_{VV}-G^{*}I_{VH}}{I_{VV}+2^{*}G^{*}I_{VH}},$$

where *G* is the spectrofluorometer’s sensitivity factor given by:

$$G=\frac{I_{HV}}{I_{HH}},$$

where *I*_*HV*_ and *I*_*HH*_ are measured using horizontally polarized excitation and vertically and horizontally polarized emission, respectively. *G* value was automatically recalculated at each points of the anisotropy measurements.

### Drugs and Chemicals

Myr from *Mycelia sterilia* (PubChem CID: 643894) (Sigma, St. Louis, MO, United States) was dissolved in DMSO (PubChem CID: 679) to obtain 5 mM stock solution. Further dilutions were made with ECS or DMSO to reach the final concentrations of 100 nM or 1 mM, respectively. SMase from *Bacillus cereus* (PubChem CID: 6476900) was purchased from Sigma in a glycerol buffered solution, and further dilutions were made with ECS or saline to reach the concentrations of 30 or 50 mU. CAPS (PubChem CID: 1548943) was purchased from Sigma and diluted with saline from a 10 mg/ml stock solution of 10% ethanol (PubChem CID: 702), 10% Tween 80 (PubChem CID: 5284448) in saline. RTX (PubChem CID: 5702546) was purchased from Sigma and was dissolved in ethanol to yield a 1 mg/ml stock solution. Further dilutions were made with saline to reach final concentrations of 30 and 0.1 µg/ml, respectively. Laurdan (PubChem CID: 104983) (Sigma) was dissolved in DMSO to obtain 10 mM stock solution, and further dilution was made with ECS to reach final concentration of 40 µM. Formalin (PubChem CID: 712) - dilution was made with PBS (PubChem CID: 24978514) to reach final concentration of 2.5% - was prepared from a 6% buffered formaldehyde stock solution (Molar Inc. Hungary).

### Statistical Analysis

Fluorescence spectroscopy measurements were performed with four samples per group. All of the animal experiment data are presented as means ± SEM of six animals per group. Statistical analysis was performed by repeated measurement (RM) two-way ANOVA - the investigated factors were the pretreatment, time and their interaction (pretreatment x time) - with Bonferroni multiple comparisons post hoc test, in all cases *p* < 0.05 was considered statistically significant.

## Results

### Sphingomyelinase and Myriocin Reduce the Number of Capsaicin-Evoked Eye-Wipings

**FIGURE 1:**
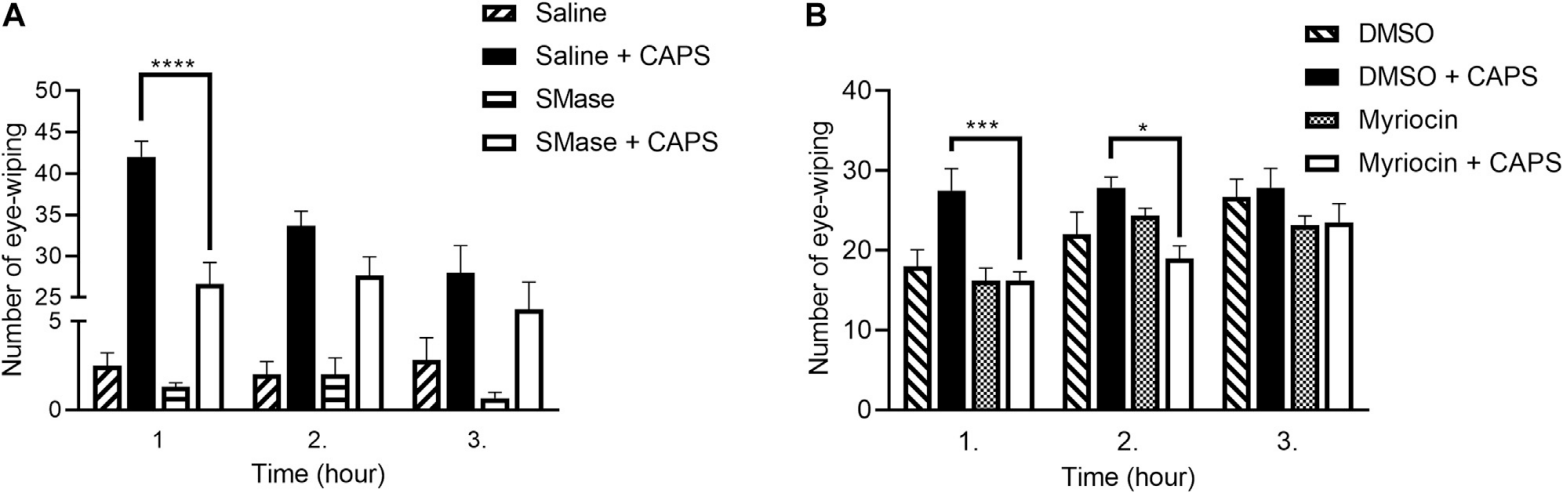
Number of the eye-wiping movements in the CAPS-evoked acute chemonociceptive reaction. 50 mU SMase **(A)** and 1 mM Myr **(B)** also reduced the number of the eye-wiping movements. Data are means ± SEM of n = 6 animals/group. The corresponding F values of the pretreatment, time and their interaction were F_(3,20)_ = 292.3; F_(2,40)_ = 5.240; F_(6,40)_ = 3.731 in case of SMase pretreatment and F_(3,20)_ = 8.182; F_(2,40)_ = 9.976; F_(6,40)_ = 1.598 in case of Myr pretreatment, respectively. RM-Two-way ANOVA with Bonferroni post hoc test was used for statistical analysis (*p < 0.05; ***p < 0.001; ****p < 0.0001 SMase/Myr pretreatment vs. saline/DMSO pretreatment).

The number CAPS-evoked eye-wiping movements within a 1-min period was 42.0 ± 1.8, 33.6 ± 1.7 and 28.0 ± 3.2 1, 2 and 3 h after local saline-pretreatment, respectively, in the control group showing desensitization in response to repeated CAPS administration. SMase pretreatment significantly decreased the number of wiping in the first hour, the corresponding values were 26.6 ± 2.5, 27.6 ± 2.2 and 24.6 ± 2.1 (Figure 1A).

Since Myr was dissolved in DMSO, this vehicle control was used in this experimental series. We also tested the DMSO in saline-pretreated animals compared to the DMSO-pretreated ones and we did not find significant difference (data not shown). In DMSO-pretreated animals CAPS administration induced 27.5 ± 2.8; 27.8 ± 1.4; 27.8 ± 2.4 eye wiping movements in the first, second and third hour, respectively. Myr pretreatment significantly diminished the number of eye-wipings in the first and second hour, these values were 16.2 ± 1.1; 19.0 ± 1.5; 23.5 ± 2.3, respectively (Figure 1B).

### Sphingomyelinase and Myriocin Decrease the Resiniferatoxin-Induced Thermal Allodynia, and Sphingomyelinase Abolish Mechanical Hyperalgesia

**FIGURE 2:**
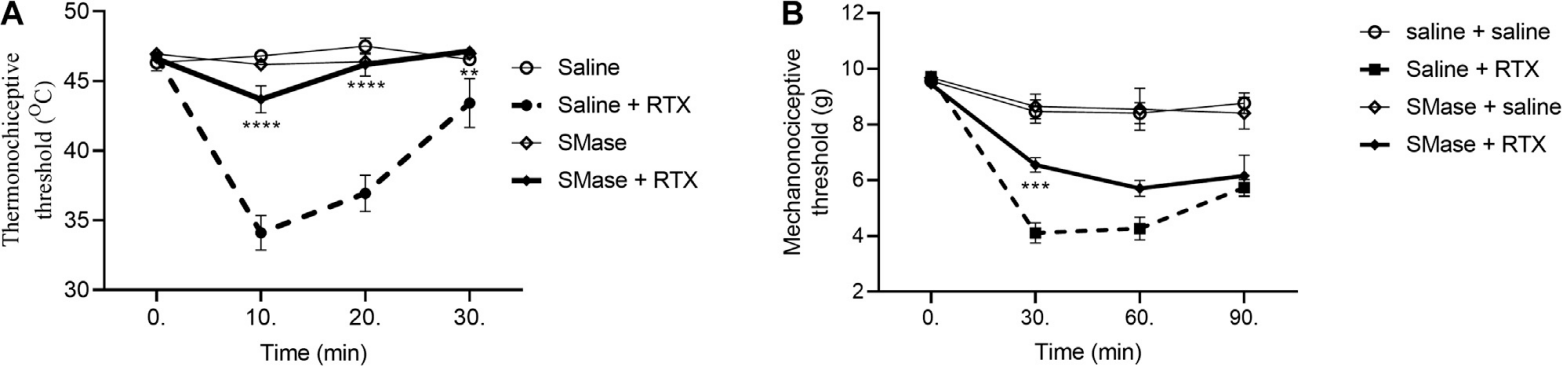
Effect of 50 mU SMase in the RTX-induced thermal allodynia **(A)** and mechanical hyperalgesia **(B)**. Data are means ± SEM of n = 6 animals/group. Dashed and solid curves represent the saline and SMase pretreatments after RTX injection, respectively. The corresponding F values of the pretreatment, time and their interaction were F(3,20) 94.61; F(3,60) 19.76; F(9,60) 12.17 in the thermal allodynia and F(3,20) 22.88; F(3,60) 64.75; F(9,60) 9.859 in themechanical hyperalgesia respectively. RM-Two-way ANOVA with Bonferroni post hoc test was used for statistical analysis (**p < 0.01; ***p < 0.001; ****p < 0.0001 SMase-pretreated group vs. saline-pretreated group).

The baseline heat threshold values of untreated mice were between 44 and 49°C. RTX induced 27.0 ± 2.8%; 20.9 ± 3.0% and 7.1 ± 3.8% drop of the thermonociceptive thresholds 10th, 20th and the 30th min after its intraplantar injection in the saline-pretreated control group. SMase pretreatment abolished the thermal allodynia in all measurement points with a decrease by 6.2 ± 1.8%; 0.8 ± 1.8% and an increase by 1.2 ± 0.4%, respectively (Figure 2A). The basal mechanonociceptive thresholds of intact mice were in a range of 9 and 10 g. RTX-evoked drop of the mechanonociceptive threshold values were 58.1 ± 3.9%, 55.6 ± 4.2%, 42.6 ± 2.5% 30, 60, and 90 min after the injection in the saline-pretreated control. SMase significantly alleviated the mechanical hyperalgesia in the 30th min, the respective values were 30.7 ± 2.5%, 39.4 ± 3.3%, 34.5 ± 8.2% (Figure 2B).

**FIGURE 3:**
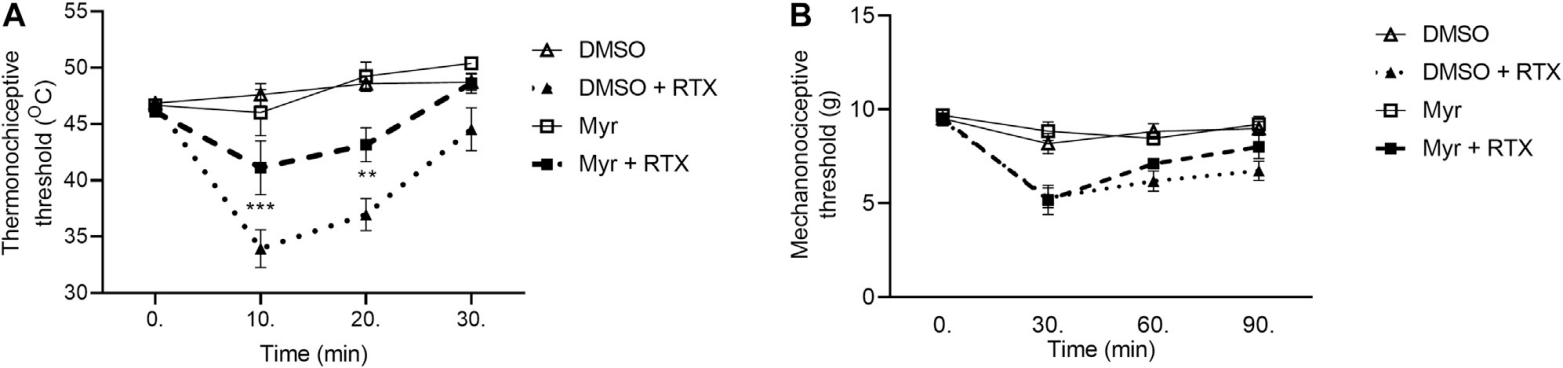
Effect of 1 mM Myr in the RTX-induced thermal allodynia **(A**) and mechanical hyperalgesia **(B)**. Data are means ± SEM of n = 6 animals/group. Dotted and dashed curves represent the DMSO and Myr pretreatments after RTX injection, respectively. The corresponding F values of the pretreatment, time and their interaction were F(3,20) 20.80; F(3,60) 19.62; F(9,60) 6.617 in the thermal allodynia and F(3,20) 11.95; F(3,60) 35.96; F(9,60) 4.736 in the mechanical hyperalgesia respectively. RM-Two-way ANOVA with Bonferroni post hoc test was used for statistical analysis (**p < 0.01; ***p < 0.001 Myr-pretreated group vs. DMSO-pretreated group).

In case of Myr, the thermo- and mechanonociceptive threshold values of intact mice were between 44 and 49°C, 8 and 10 g, respectively. DMSO, as solvent of Myr was tested in saline-pretreated animals in contrast with DMSO-pretreated animals and significant desensitization effect was not revealed (data not shown). After RTX injection the thermonociceptive threshold in the DMSO-pretreated group decreased by 27.5 ± 3.2%; 21.0 ± 3.2% and 4.9 ± 3.8% in the 10th, 20th and 30th min, respectively, while Myr-pretreated group values decreased by 10.7 ± 5.2%; 6.3 ± 3.0% after 10 and 20 min its injection and increased by 5.5 ± 1.4% after 30 min. Myr pretreatment diminished the thermal allodynia in the 10th and 20th but not in the 30th min (Figure 3A). RTX-evoked mechanical hyperalgesia in the DMSO-pretreated control group were 43.9 ± 4.8%; 34.7 ± 4.3%; 28.6 ± 4.7% on the measurement points, compared to the Myr-pretreated group, these values were similar: 45.2 ± 8.7%; 25.4 ± 3.2%; 15.9 ± 6.9%. Myr pretreatment did not altered the mechanical hyperalgesia at any time point (Figure 3B).

### Sphingomyelinase Diminish Formalin-Evoked Acute Nocifensive Behaviors

**FIGURE 4:**
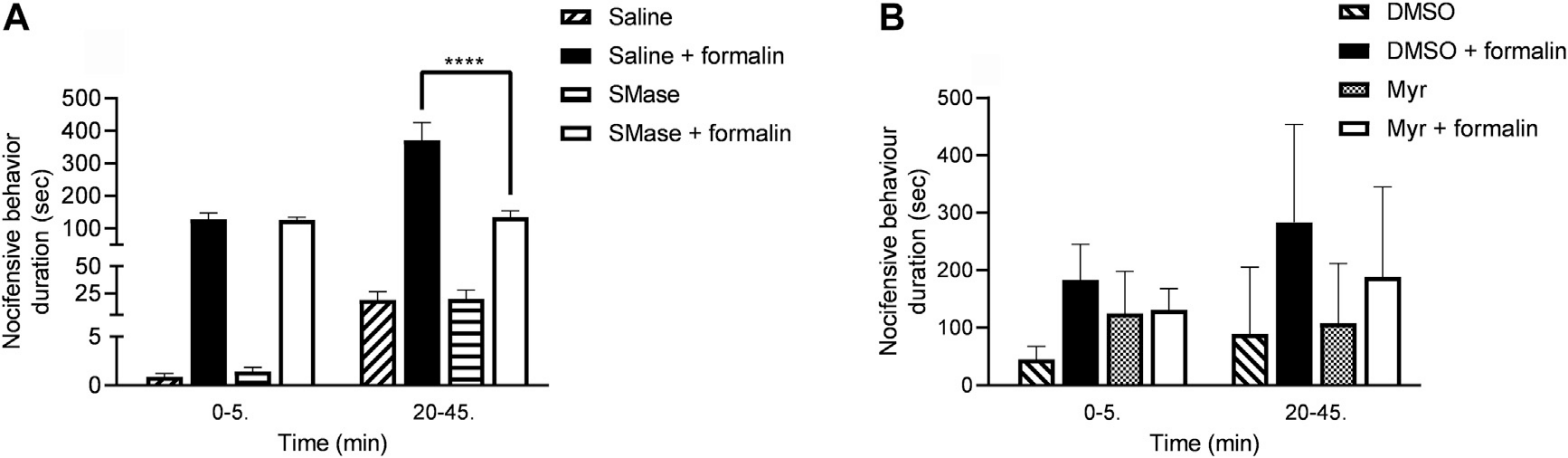
Effects of 50 mU SMase **(A)** and 1 mM Myr **(B)** in the formalin-evoked acute nocifensive behaviors reaction. Data are means ± SEM of n = 6 animals/group. RM-Two-way ANOVA with Bonferroni post hoc test was used for statistical analysis (****p < 0.0001 SMase/Myr pretreatment vs. saline/DMSO pretreatment). The corresponding F values of the pretreatment, time and their interaction were F_(3,20)_ = 37.02; F_(1,20)_ = 39.06; F_(3,20)_ = 24.68 in case of SMase pretreatment and F_(3,20)_ = 4.318; F_(1,20)_ = 3.032; F_(3,20)_ = 0.8396 in case of Myr pretreatment, respectively.

The durations of formalin-induced paw lickings, liftings and shakings in the saline-pretreated control group were 127.2 ± 18.5 s and 371.1 ± 54.4 s in the first and second phases, respectively. SMase-pretreatment did not influence the acute chemonocifensive behavior in first phase due to direct activation of TRPA1 receptors on the sensory nerve endings, but induced significant inhibition in the second phase related to the acute neurogenic inflammatory reaction. The corresponding results were the following in the first and second phases, respectively: 125.9 ± 8.0 and 134.1 ± 20.1 s (Figure 4A).

In case of Myr, DMSO as its solvent was tested in saline-pretreated animals in contrast to DMSO-pretreated animals, and significant differences in the two groups was not found (data now shown). The formalin-evoked nocifensive behavior durations in the first phase were 183.1 ± 25.3 and 131.0 ± 15.2 s in the DMSO-pretreated control group and Myr-pretreated group, respectively. The durations of the second phase were 283.4 ± 69.4 and 188.7 ± 63.8 s in the DMSO control and Myr-pretreated animals. Myr had some biological effect, however this was not significant (Figure 4B).

### Myriocin Changes the Membrane Polarity

**FIGURE 5:**
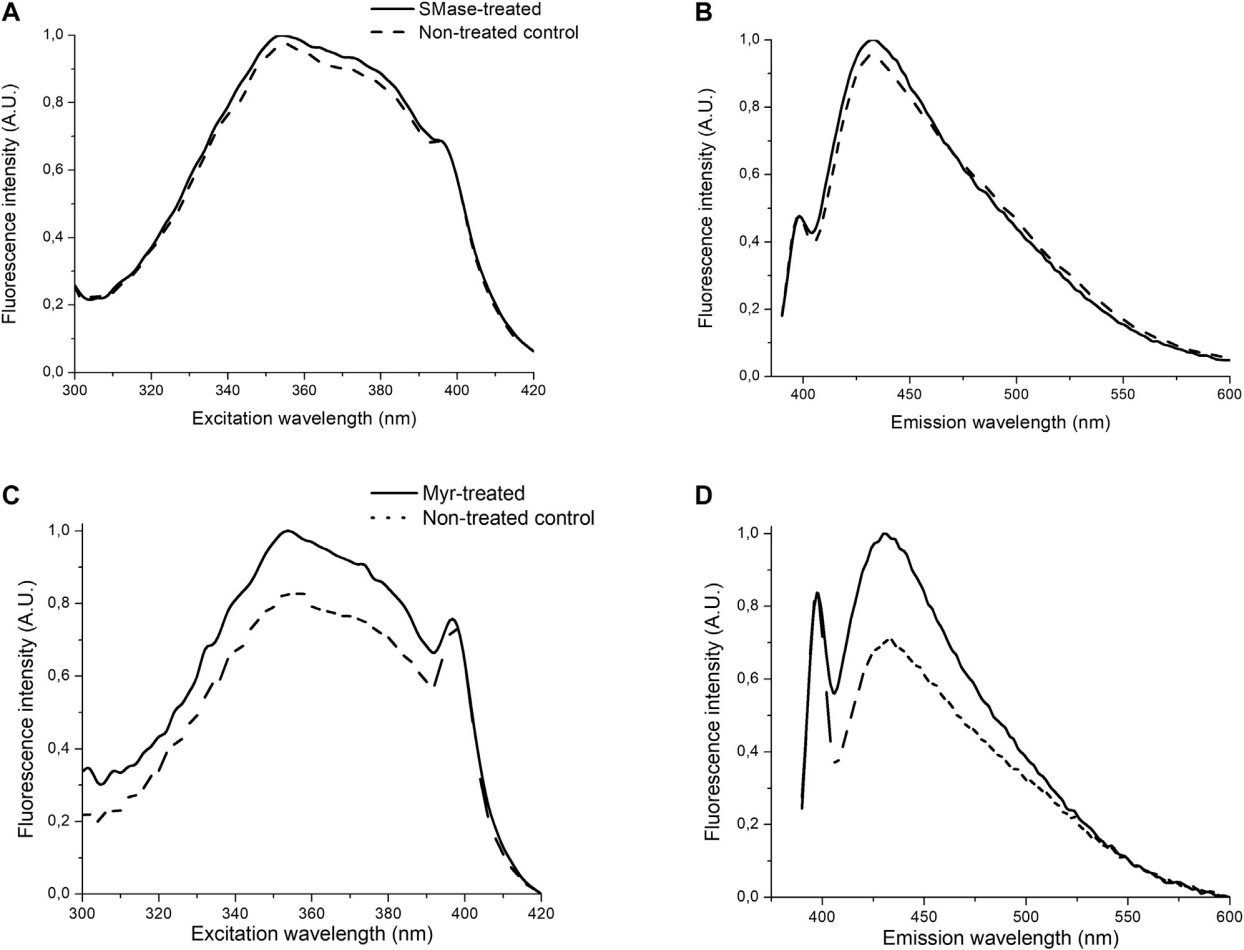
Effects of 30 mU SMase or 100 nM Myr on normalized excitation- **(A,C)** or emission spectra **(B,D)** of Laurdan. Solid and dashed/dotted curves represent the values measured on the SMase/Myr-treated and non-treated control samples, respectively. Excitation wavelength: 350 nm, emission wavelength: 460 nm.

**FIGURE 6:**
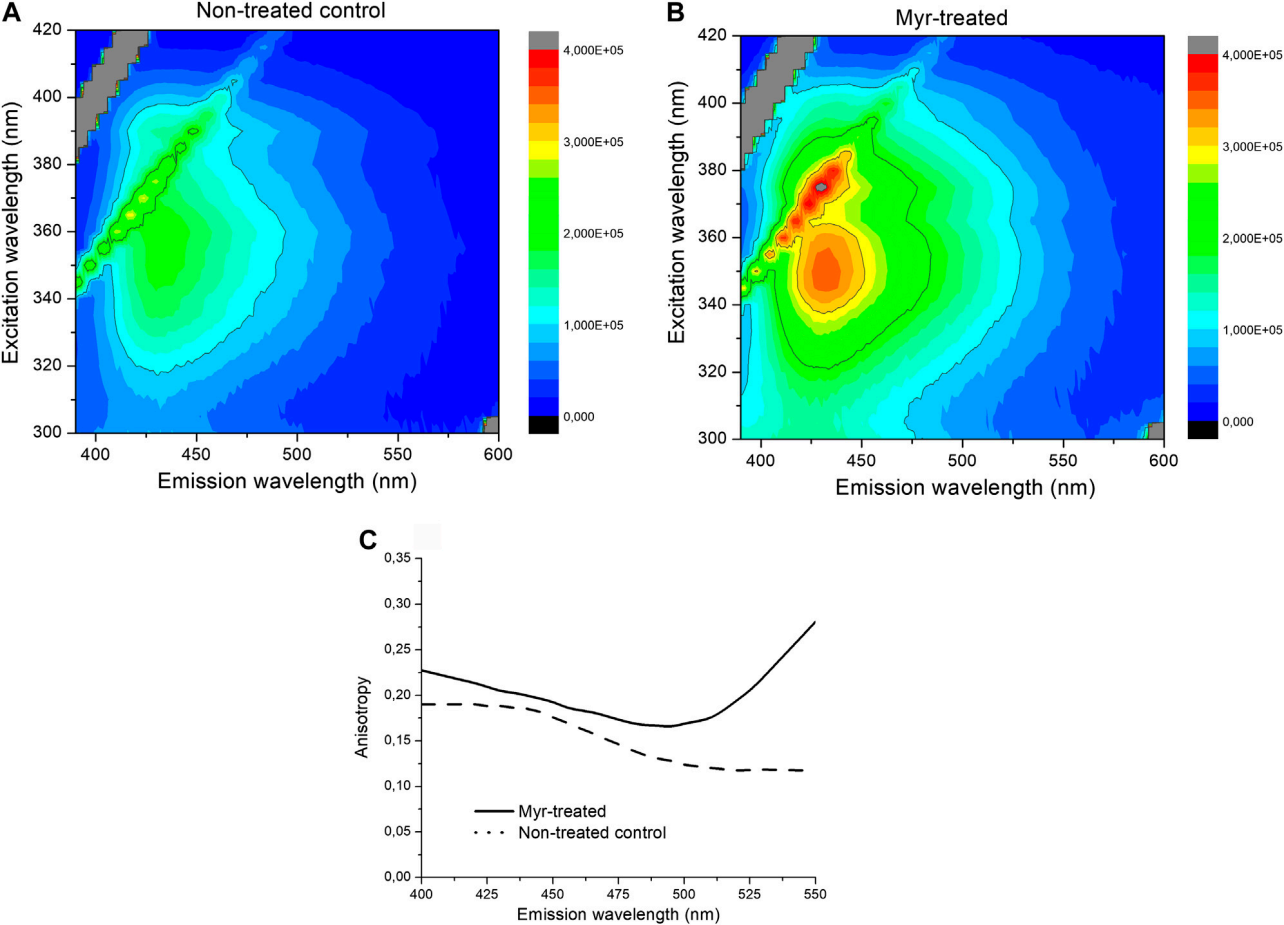
Excitation-emission matricies of non-treated control **(A)** and 100 nM Myr-treated **(B)** samples, respectively. Emission anisotropy spectra of 100 nM Myr-treated (solid) and non-treated control (dotted) samples.

SMase treatment did not influence the fluorescence spectroscopy picture as compared to the non-treated control. Spectral shift, broadening or changes in the shape of the spectra or intensity were not detected, as shown by no signal alterations of Laurdan. No changes were detected in the steady-state emission anisotropy measurements and transition from liquid-ordered to -disordered phase between the SMase-treated and non-treated cells (Figures 5A,B). In contrast to SMase, Myr treatment significantly modified the membrane microenvironment. The fluorescence emission was stronger in the Myr-treated samples than in the non-treated ones as shown by both excitation and emission spectra (Figures 5C,D). Excitation-emission matrices on the entire spectral region of Laurdan fluorescence also showed that the Myr-treated samples had higher fluorescence intensity than the non-treated control samples (Figures 6A,B). Emission anisotropy values of Myr-treated samples were significantly higher on the whole spectral range (Figure 6C).

## Discussion

We present here the first data on the analgesic effect of lipid raft disruption by SMase-induced SM hydrolization (Kiyokawa et al., 2005; Kobayashi et al., 2006) or Myr-induced glycosphingolipid synthesis blockade (Miyake et al., 1995). We proved that SMase and Myr decrease CAPS-evoked eye-wipings, as well as RTX-induced thermal allodynia, furthermore, SMase decreases the RTX-induced mechanical hyperalgesia and duration of the formalin-evoked -acute nocifensive behavior.

SMase and Myr significantly decreased TRPV1 activation-evoked eye-wiping movements by 37 and 41%, respectively. The decreasing response to repeated CAPS instillation was due to TRPV1 receptor desensitization (Sharma et al., 2013). Furthermore, SMase almost abolished TRPV1 stimulation-induced thermal allodynia and significantly reduced mechanical hyperalgesia. Myr had significant inhibitory effect on thermal allodynia induced predominantly by peripheral sensitization mechanisms, but not mechanical hyperalgesia involving central sensitization processes as well (Pan et al., 2003). TRPA1 activation-evoked acute neurogenic inflammatory nocifensive reactions were also significantly diminished by SMase (by 64%), while only non-significant decreasing tendency was induced by Myr.

These novel *in vivo* findings are well supported by our previous *in vitro* results demonstrating the ability of SMase and Myr to significantly and concentration-dependently inhibit TRPV1 receptor activation both on TRPV1 receptor-expressing cell line and primary cultures of trigeminal sensory neurons, similarly to MCD (Szőke et al., 2010). Furthermore, similar inhibitory actions of these lipid raft disruptors were described on the TRPA1, TRP Melastatin 8, but not the TRP Melastatin 3 cation channels (Sághy et al., 2015).

We provide the first direct evidence by fluorescence spectroscopy for the ability of Myr to induce transition from liquid-ordered to liquid-disordered phase indicating cholesterol depletion in the plasma membrane. Higher fluorescence intensity means less non-radiative processes of Laurdan and higher anisotropy reflects to more restricted motion of Laurdan after Myr treatment. These findings together indicate a more compact, closed membrane structure around Laurdan molecules. In contrast, SMase treatment did not influence the membrane polarity. We have previously proved that fluorescence spectroscopy is an appropriate technique to determine the plasma cholesterol content by comparing this method with the conventional filipin staining after MCD treatment in the same cell cultures (Sághy et al., 2015).

The formation of lipid rafts in the plasma membrane depends on the interaction of SM and cholesterol (Simons and Ikonen, 1997; Ridgway, 2000). Cholesterol content of the lipid membrane does not affect significantly the biosynthesis of SMs being involved in a variety of essential cellular functions (Ridgway, 2000). It has been reported that SM and cholesterol synthesis are independent processes in cultured human intestinal cells, but the amount of cholesterol and SM regulate their synthesis rates (Chen et al., 1993). It has been described that orally administered Myr decreases not only SM, but also cholesterol levels of the small intestinal epithelial cell plasma membrane (Li et al., 2009).

Although results of *in vitro* experiments provided evidence, that lipid raft disruption inhibited the activation mechanisms of TRP channels (Szőke et al., 2010; Sághy et al., 2015), there are only few recent animal experiments to prove this phenomenon *in vivo*. Cholesterol depletion by MCD induced antinociception in RTX-induced neuropathy in the mouse through phosphatidylinositol 4,5-bisphosphate hydrolysis (Lin et al., 2019). Furthermore, MCD and a novel carboxamido-steroid compound were able to exert antinociceptive effects by decreasing the activation of TRPV1 and TPRA1 ion channels in mice in distinct pain mechanisms (Horváth et al., 2020). The mechanical hyperalgesia induced by PGE2, but not cyclopentyladenosine was attenuated by MCD injection into the rat paw, suggesting that PGE2-evoked hyperalgesic effect is dependent on the lipid raft integrity (Ferrari and Levine, 2015). Complete Freund’s adjuvant-induced thermal and mechanical hyperalgesia was attenuated by both local and systemic administration of random methylated β-cyclodextrins (RAMEB) in rats. The authors suggest that RAMEB capture the prostaglandin content and then decrease the inflammatory pain which might be a novel anti-inflammatory and analgesic tool (Sauer et al., 2017). In a very recent paper, the role of another components of lipid rafts, the gangliosides have been discussed in pain mechanisms (Sántha et al., 2020). Intraplantar injection of the ganglioside GT1b induced nociceptive responses and augmented formalin-evoked nocifensive reactions. Nevertheless, sialidase injection cleaving sialyl residues from gangliosides is able to diminish the nociceptive responses (Watanabe et al., 2011; Sántha et al., 2020).

The role of sphingolipids in pain sensation via the modulation of TRP channel activation is poorly understood and we had only *in vitro* experimental results (Szőke et al., 2010; Sághy et al., 2015). The present results are the first *in vivo* data providing clear evidence that sphingolipids play an important role in both TRPV1 and TRPA1-evoked nocifensive behaviors and hyperalgesia. Intraplantar pretreatments by both SMase and Myr inhibited thermal allodynia in the acute neurogenic inflammation model that develop mainly by peripheral sensitization of the sensory nerves. Furthermore, SMase injection into the paw even decreased mechanical hyperalgesia induced by both peripheral and central sensitization mechanisms (Pan et al., 2003), demonstrating a stronger inhibitory effect on the pseudo unipolar primary sensory neuron as compared to Myr. This observation together with the fact that SMase does not influence the membrane cholesterol composition support our conclusion on the key importance of sphingolipids over cholesterol in lipid raft integrity around TRPV1 and TRPA1 and consequent sensory nerve activation.

These *in vivo* results strongly suggest that membrane sphingolipid modification particularly by SMase might open novel analgesic opportunities. This innovative approach potently inhibits the activation of the sensory nerves via targeting pain sensing structures including TRPV1 and TRPA1. This might be particularly promising since it is more likely to inhibit the pathological activation, but not the physiological functions of TRPV1 and TRPA1. Moreover, other main advantages are the opportunity for local administration and its rapid onset of action. Therefore, SMase seems to be a promising therapeutic tool with a good side effect profile (e.g. it could avoid the hyperthermic side effects of TRPV1 antagonists).

## Data Availability Statement

The original contributions presented in the study are included in the article/Supplementary Material, further inquiries can be directed to the corresponding author.

## Ethics Statement

The animal study was reviewed and approved by Ethics Committee on Animal Research of Pécs University.

## Author Contributions

Conceptualization ÉSz and JS; methodology ÁH, BK, TB-S, MP, ÉSá, GM, JE, AS; formal analysis ÁH; investigation ÁH, BK, TB-S, MP, ÉSá, GM, JE, AS; resources ZH, ÉSz; writing—original draft preparation, ÁH; writing—review and editing ÉSz, ZH; visualization ÁH; supervision ÉSz, ZH; project administration ÁH, TB-S; funding acquisition ZH, ÉSz.

## Funding

This work was supported by the National Brain Research Program 2017-1.2.1-NKP -2017-00002 (NAP-2; Chronic Pain Research Group). We acknowledge the grant of the Hungarian Government (GINOP-2.3.2-15-2016-00050, EFOP-3.6.2-16-2017-00006 and EFOP-3.6.2-16-2017-00008). ÉSz and ÉSá were supported by the János Bolyai Research Scholarship of the Hungarian Academy of Sciences. The University of Pécs is acknowledged for a support by the 17886-4/23018/FEKUTSTRAT excellence grant. MP was supported by the New National Excellence Program of the Ministry of Human Capacities ÚNKP-18-4. ÉSz and ÉSá were supported by the New National Excellence Program of the Ministry of Human Capacities ÚNKP-18-4 and New National Excellence Program of the Ministry for Innovation and Technology ÚNKP-19-4 grant. ÁH was supported by the Gedeon Richter’s Talentum Foundation.

## Conflict of Interest

The authors declare that the research was conducted in the absence of any commercial or financial relationships that could be construed as a potential conflict of interest.

## **Abbreviations**

CAPS, capsaicin; CGRP, calcitonin gene-related peptide; CHO, Chinese hamster ovary; ECS, extracellular solution; Laurdan, 6-dodecanoyl-N,N-dimethyl-naphthylamine; MCD, methyl β-cyclodextrin; Myr, myriocin; PGE2, prostaglandin E2; RAMEB, random methylated β-cyclodextrins; RM, repeated measurement; RTX, resiniferatoxin; TRP, Transient Receptor Potential; TRPA1, Transient Receptor Potential Ankyrin 1; TRPC1, Transient Receptor Potential Canonical 1; TRPC3, Transient Receptor Potential Canonical 3; TRPV1, Transient Receptor Potential Vanilloid 1.
